# Effect of Newly Synthesized Structures of Peptides on the Stability of the Monolayers Formed

**DOI:** 10.3390/ijms24054318

**Published:** 2023-02-21

**Authors:** Iwona Golonka, Katarzyna E. Greber, Bartłomiej M. Szyja, Patrycja P. Petrus, Jakub E. Pucułek, Witold Musiał

**Affiliations:** 1 Department of Physical Chemistry and Biophysics, Wroclaw Medical University, Borowska 211A, 50-556 Wrocław, Poland; 2 Department of Physical Chemistry, Faculty of Pharmacy, Medical University of Gdańsk, Al. Gen. J. Hallera 107, 80-416 Gdańsk, Poland; 3 Faculty of Chemistry, Wrocław University of Science and Technology, ul. Gdańska 7/9, 50-344 Wrocław, Poland

**Keywords:** Langmuir monolayer, compression isotherm, peptides, TG/DTG, DSC, molecular dynamics simulations

## Abstract

The aim of the study was to evaluate the effect of the peptide structure (WKWK)_2_-KWKWK-NH_2_, P4 (C12)_2_-KKKK-NH_2_, P5 (KWK)_2_-KWWW-NH_2_, P6 (KK)_2_-KWWW-NH_2_ on their physicochemical properties. The thermogravimetric method (TG/DTG) was used, which made it possible to observe the course of chemical reactions and phase transformations occurring during the heating of solid samples. Based on the DSC curves, the enthalpy of the processes occurring in the peptides was determined. The influence of the chemical structure of this group of compounds on their film-forming properties was determined using the Langmuir–Wilhelmy trough method and was followed by molecular dynamics simulation. Evaluated peptides showed high thermal stability and the first significant mass loss occurred only at about 230 °C and 350 °C. The analysis of the compressibility coefficient of individual peptides indicates that all formed peptide monolayers were in the expanded liquid phase. Their maximum compressibility factor was less than 50.0 mN/m. Its highest value of 42.7 mN/m was achieved in a monolayer made of P4. The results obtained in molecular dynamic simulation indicate that non-polar side chains played an important role in the properties of the P4 monolayer, and the same applies to P5, except that a spherical effect was observed here. A slightly different behavior was observed for the P6 and P2 peptide systems, where the type of amino acids present had an influence. The obtained results indicate that the structure of the peptide affected its physicochemical and layer-forming properties.

## 1. Introduction

Compounds containing amino acid residues are currently one of the most frequently synthesized and investigated groups of compounds. Due to their ability to self-assemble, they are used to produce nanomaterials with high biocompatibility. They have found application, for example, as a way of delivering drugs to cells [1,2,3]. Thanks to the proven antibacterial properties of some surfactants, they are a promising group that can help fight antibiotic resistance in the near future [4,5]. The ratio of cationic and hydrophobic residues determines the activity of antimicrobial peptides (AMPs). The cationic residues include amino acids such as arginine (R), lysine (K) or histidine (H). Their role is to mediate reactions with negatively charged bacterial lipids. Hydrophobic residues containing tryptophan—W, phenylalanine—F and leucine—L are involved in connecting with cell membranes and determine their damage [6]. Polar cationic residues facilitate the solubilization of molecules in water, while lipophilic residues enable localization in lipid micelles [7,8]. Some factors may alter the properties of the monolayers, e.g., according to Wang et al. the quantum dots may reduce the compressibility of the monolayer at increased temperature [9].

The work published in 1891 by Pockels on the change of surface tension and thickness of the compressed oil layer on the water surface provided the basis for the characterization of Langmuir layers at the water/air interface [10]. At the beginning of the 20th century, Irving Langmuir improved the measurement method proposed by Agnes Pockels and designed a device for measuring the surface pressure of a monolayer spread on the water surface, the so-called Langmuir trough. Langmuir proved the monomolecular nature of the oil film applied to the water surface and was the first to propose a model of the orientation of amphiphilic molecules at the water/air interface [11,12,13]. The layers thus defined were later called Langmuir monolayers, which provide an excellent model for studying biological systems. Moreover, it was proved that the properties of biological membranes and model Langmuir monolayers, formed by phospholipid molecules, compressed to a surface pressure of ~32.5 mN/m, are similar [14]. Therefore, the Langmuir trough is often used in biophysical field of medicine and drug chemistry. In addition to traditional Langmuir monolayers, there are other complementary surface-sensing techniques, such as surface electrical potential measurements [15], Brewster angle microscopy (BAM, for texture visualization and layer thickness measurement) [16], and polarization-modulated infrared reflection–absorption spectroscopy [17].

**Figure 1:**
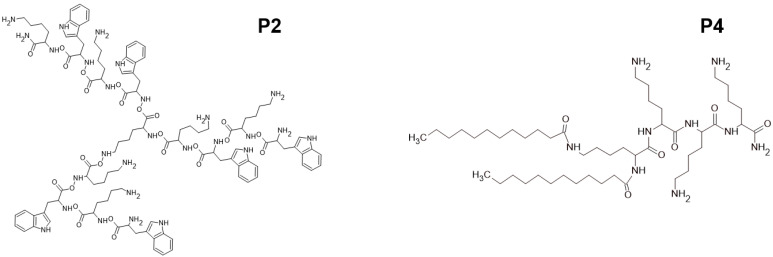

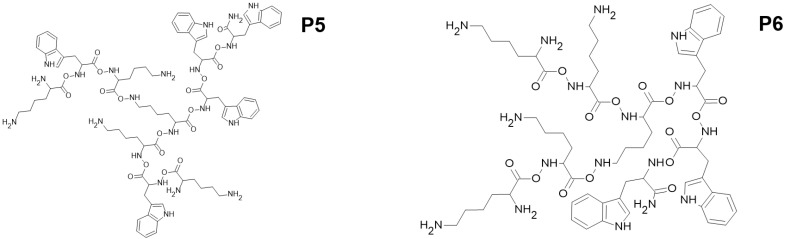
Structures of tested peptides P2, P4–P6. P2, P4–P6 are the abbreviations of the evaluated peptides described in the text [18].

From the research we have published so far, we know that the following peptides: P1 (WK)_2_-KWK-NH_2_, P2 (WKWK)_2_-KWKWK-NH_2_, P3 (WR)_2_-KWR-NH_2_, P4 (C12)_2_-KKKK-NH_2_, P5 (KWK)_2_-KWWW-NH_2_, P6 (KK)_2_-KWWW-NH_2_ exhibit antioxidant properties. P2, P4, P5 and P6 compounds exhibit antimicrobial activity against *S. aureus*. Peptide 2 is highly effective against *S. aureus*. Sorption of P2 and P4–P6 on the polymer-bacterial cellulose (BC) produced by *Komagateibacter xylinu* confirmed the prospective topical application of these peptides on the BC carrier. The mentioned compounds had no cytotoxic activity against fibroblast lines [18]. So far, only peptide 4 has been shown to have antifungal activity (*Candida albicans*, *Candida tropicalis*, *Aspergillus niger*) and has antimicrobial activity against gram-positive bacteria (*Staphylococcus epidermidis*, *Bacillus subtilis*, *Enterococcus faecalis*) as well as against gram-negative bacteria (*Escherichia coli*, *Klebsiella pneumonia*, *Pseudomonas aeruginosa*) [19]. The structures of the P2 and P4–P6 peptides are shown in Figure 1.

The aim of the presented work was to assess the impact of the structure of four selected peptides P2, P4–P6 on their physicochemical properties determined on the basis of thermogravimetric analysis (TG/DTG) and differential scanning calorimetry (DSC) of solid samples, and to prepare respective monolayers from chloroform–methanol solutions in the Langmuir–Wilhelmy through. The investigations were based on the performance of compression isotherms and hysteresis in which the change of surface pressure versus area per molecule was evaluated. The compressibility modulus was used to determine the physical state of the monolayers made of peptides composed of the amino acids tryptophan and lysine. Molecular dynamics (MD) simulation was used to understand the interactions between the individual peptides that make up the monolayer. The collected results may enable determination of the usefulness of applied compounds in varied forms.

## 2. Results

### 2.1. Characterization of Compounds Using Thermogravimetric and Differential Scanning Calorimetry

**Figure 2:**
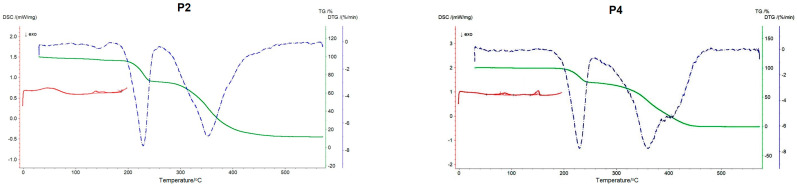

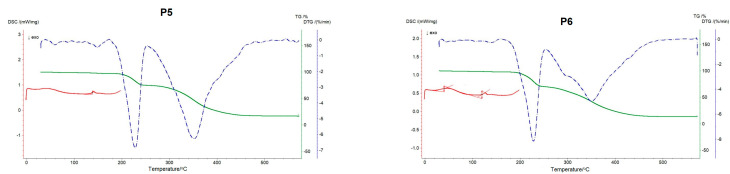
TG/DTG and DSC curves of peptide P2, P4–P6: TG (–), DTG (–), DSC (–).

Thermal analysis methods were used to study chemical reactions and phase transitions that occurred when the substance was heated or cooled. The measurement of the mass difference from the TG curve gives information about changes in the composition of the sample, thermal stability of the substance and parameters. Figure 2 show the TG/DTG and DSC curves of the tested peptides. The curves are color coded as follows TG (–), DTG (–), DSC (–). The TG curve of the distribution of samples P2, P4-P6 shows the mass loss [mg]; the DTG curve makes it easy to determine the start, end and maximum each reaction.

The data determined by the TG and DTG methods are summarized in Table 1. In the case of the P2 peptide, in the temperature ranged from 25 to 145 °C, there were two weight losses of the sample responsible for water evaporation. Around the temperature of 230 °C in all mixtures there was a second weight loss of more than 20%, and in the range of 345–352 °C the third weight loss of more than 50%. The exception iwa P4, where the third T_max_ occurred at 406.3 °C.

The DSC method is a direct measurement of the heat generated by chemical reactions and various physical processes. The respective plots present the amount of heat delivered as a function of temperature. Based on DSC studies (Table 2), we observed that all peptides underwent exothermic and endothermic transformations. In the case of P4 there were four DSC peaks, unlike in other peptides: an exothermic transformation took place as the first peak, the pattern was not observed in the remaining peptides, where the only three peaks were identified: two endothermic, and one exothermic peak, with endothermic peak as initial one.

### 2.2. Langmuir Monolayers

#### 2.2.1. Langmuir Monolayers Formed from P2, P4–P6 with Different Concentrations

**Figure 3:**
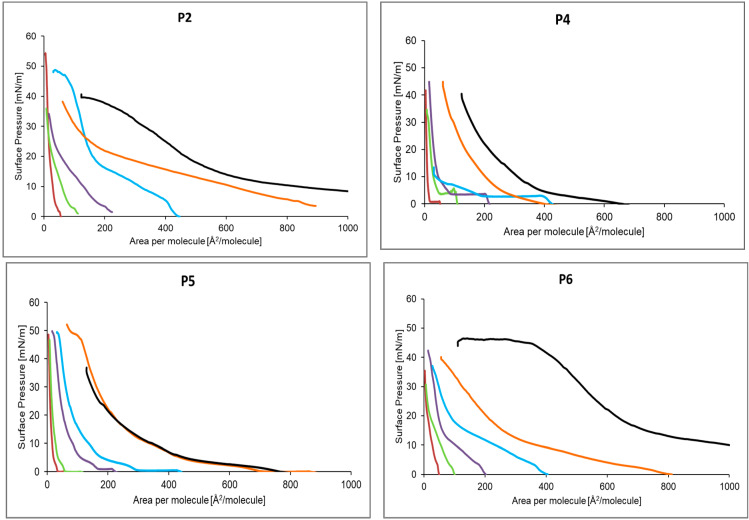
The compression isotherm of the P2, P4–P6 peptide monolayer formed on the surface of the pure liquid phase for the concentration of 1 (-), 2 (-), 3 (-), 4 (-), 5 (-), 6 (-).

Based on the obtained compression isotherms of different peptide concentrations (Figure 3), the 2.08 × 10^16^ molecules were used for further analysis, as most appropriate for the comparisons of assessed peptides (Figure 3: P2, P4–P6, D1 dilution, brown lines), due to the highest repeatability of obtained plots in three series of measurements. A lift-off value of surface area per compound molecule, for which an increase in surface pressure is observed above 0 for all compounds was within the range of 50–65 Å^2^/molecule in D1 dilutions.

#### 2.2.2. Monolayer Compression and Expansion Isotherms from P2, P4–P6

**Figure 4:**
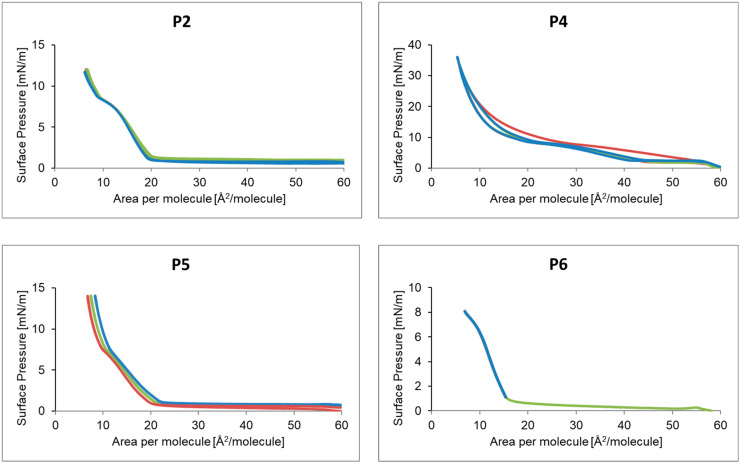
Hysteresis of the peptide P2, P4–P6 for the number of molecules equal to 2.08 10^16^. Loop 1 (-), Loop 2 (-), Loop 3 (-).

The compression and decompression isotherms of the P2, P4–P6 monolayer for a selected number of molecules of a given compound, amounting to 2.08·10^16^ are shown in Figure 4. The P2 hysteresis ran in the surface pressure range of 0–12 mN/m. The surface pressure for the first compression started to increase rapidly at 20 Å^2^/molecule. Decompression followed almost the traces of compression. The second hysteresis, compared to the first, ended with a slightly larger surface area per molecule of the tested compound. A similar situation was observed for the third loop. At a surface pressure of 6 mN/m, the curve began to flatten, which could indicate a phase transition. In the case of P4, the hysteresis was within the surface pressure range of 0–36 mN/m. The greatest increase in pressure occurred at 12 Å^2^/particle. The second compression followed the steps of the first decompression and the third followed the second. The distance between compression and decompression in successive loops decreased. The highest surface pressure was recorded at 5.5 Å^2^/particle. The P5 hysteresis was recorded for the surface pressure range from 0 to 14 mN/m. For each hysteresis, the course of decompression coincided with the course of compression. The hysteresis loop 1, 2, 3 successively was shifted towards larger surfaces per molecule. In the case of P6, the hysteresis was recorded for the surface pressure range from 0 to 8 mN/m. The maximum surface pressure was reached at 7 Å^2^/particle. The following cycles coincided with the first cycle.

#### 2.2.3. Compressibility Modulus C_s_^−1^ from P2, P4–P6

**Figure 5:**
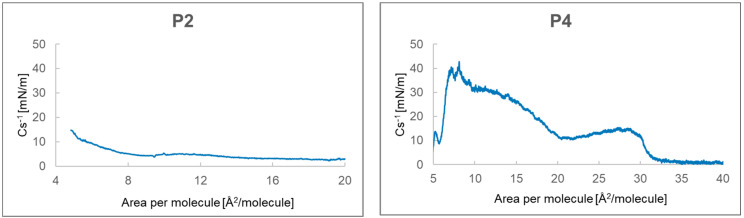

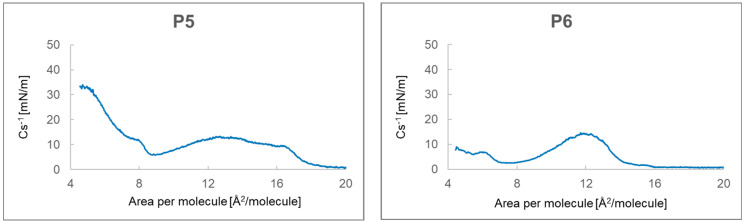
Dependence of the compressibility coefficient depending on the surface area per particle for P2, P4–P6.

Analyzing Figure 5, it can be seen that the highest value of the compressibility coefficient equal to 42.7 mN/m was achieved by P4 at the value of 8.1 Å^2^/particle. A second maximum of 15 mN/m was observed at 29 Å2/particle. The compressibility factor for P2 showed the highest value of 14.7 mN/m at 4.8 Å^2^/particle and for P5 34.0 mN/m at 4.7 Å^2^/particle. In the case of P6, at 12.1 Å2/particle, the compressibility coefficient had the highest value of 14.6 mN/m, which decreased with less surface area per particle.

#### 2.2.4. Compression Isotherms of Monolayer for Mixtures in Different Proportions P2, P4–P6

**Figure 6:**
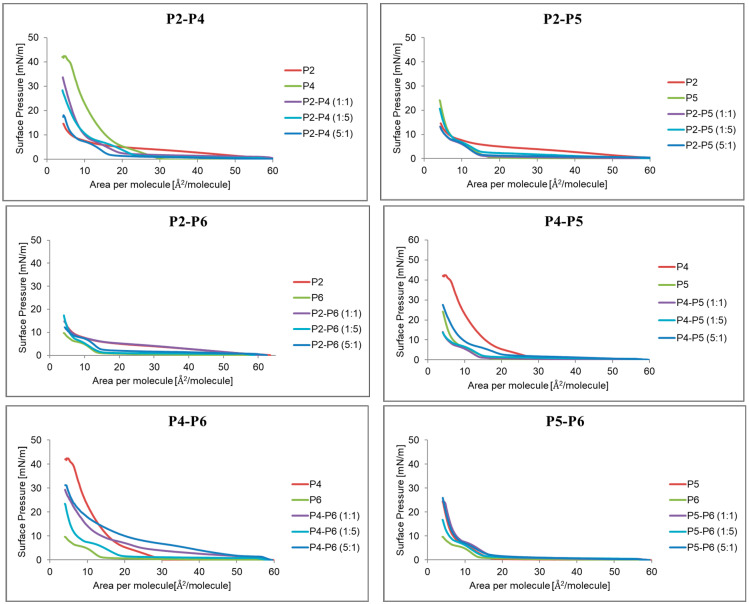
Isotherm of compression of a monolayer formed from a mixture of compounds P2, P4–P6 in different proportions on the surface of a pure liquid phase.

Figure 6 shows the compression isotherms of the systems of compounds P2, P4, P5, P6 mixed in the proportions of 1:1, 1:5 and 5:1. In the P2-P4 system, it can be seen that the predominance of P2 significantly lowered the surface pressure. On the other hand, P4 increased the surface pressure in the systems. For the P2–P5 or P2–P6 system, the changes were less visible.

#### 2.2.5. Monolayer Compression and Decompression Isotherms for Mixtures in Different Proportions P2, P4–P6

**Figure 7:**
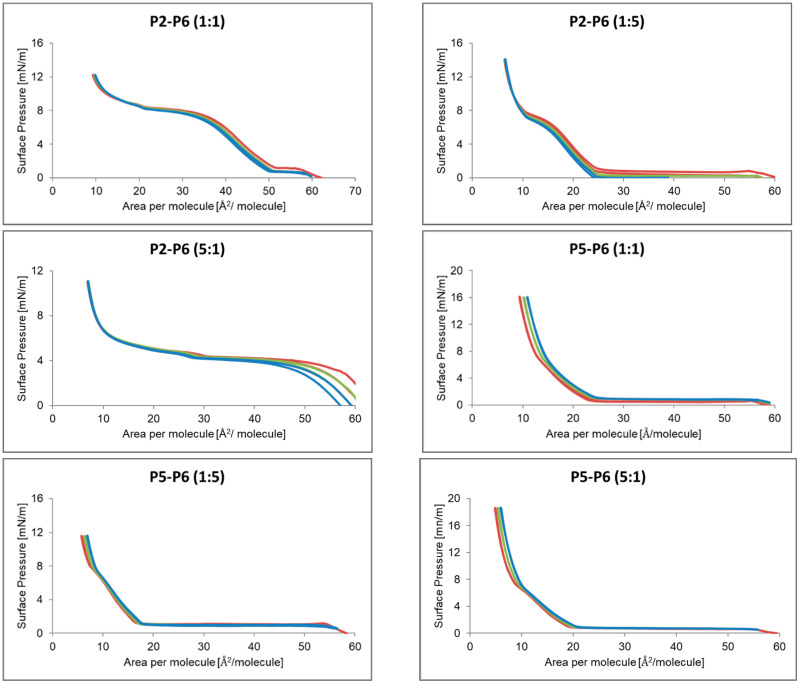
Hysteresis of the mixtures P2, P5, P6 peptide in different proportion. Loop 1 (**-**), Loop 2 (**-**), Loop 3 (**-**).

**Figure 8:**
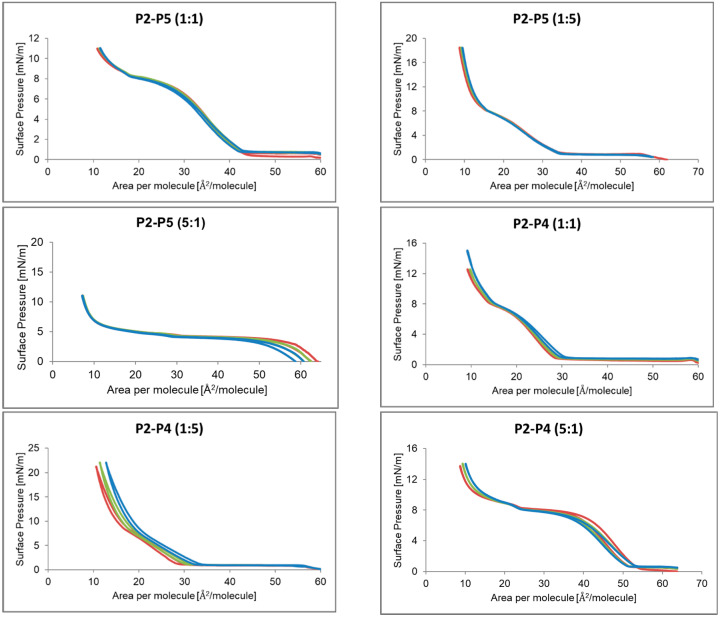
Hysteresis of the mixtures P2, P4, P5 peptide in different proportion. Loop 1 (**-**), Loop 2 (**-**), Loop 3 (**-**).

Figure 7 shows the hysteresis of the P2–P6 and P5–P6 peptides mixed in the proportions of 1:1, 1:5 and 5:1. Subsequent compression and decompression isotherm loops for the P2–P6 system have been shifted towards a smaller area per particle. The compression followed the previous decompression. This may indicate that the space between the particles decreased with each squeeze. In the P5–P6 systems, the area increased with the next hysteresis loop. It is similar in the case of the P2–P4 system presented in Figure 8.

In the P2–P5 system (Figure 8) the compression isotherm followed the decompression traces, creating almost one line. The exception here is the P2–P5 system, where the compounds were mixed in the proportions of 5:1, each successive compression started at the end of the previous decompression, with a decrease in the surface area per particle.

#### 2.2.6. Compressibility Modulus C_s_^−1^ for Mixtures in Different Proportions P2, P4–P6

**Figure 9:**
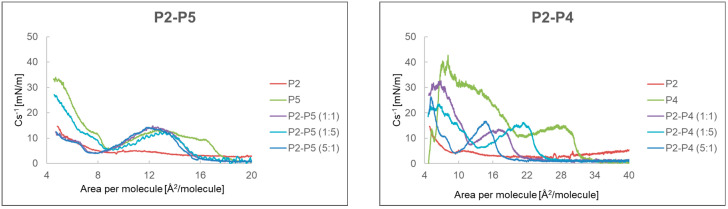

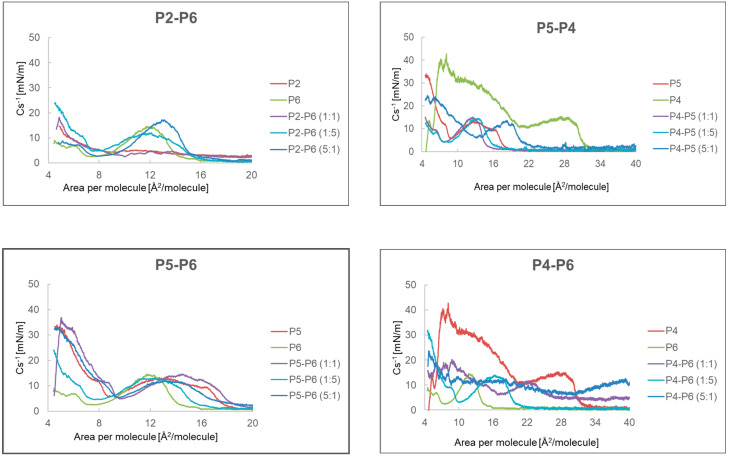
Dependence of the compressibility coefficient depending on the surface area per particle of the mixtures P2, P4–P6 peptide in different proportion.

The dependence of the compressibility factor on the surface area per particle of mixtures of P2, P4–P6 peptides in various proportions is shown in Figure 9. For the P2-P5 system, the highest compressibility factor was shown by the P5 peptide, and the addition of P2 decreased its value mainly at the first maximum of 35 mN/m to about 15 mN/m. A similar situation occurred in the P2–P4 system. Increasing the amount of P2 resulted in a shift of the second maximum towards smaller values of area per molecule. In the P5–P4 and P4–P6 systems, the P4 compound increased the value of the compressibility coefficient, while increasing the amount of P5 or P6 caused both maxima to shift to the left.

### 2.3. Molecular Dynamics Simulations

**Figure 10:**
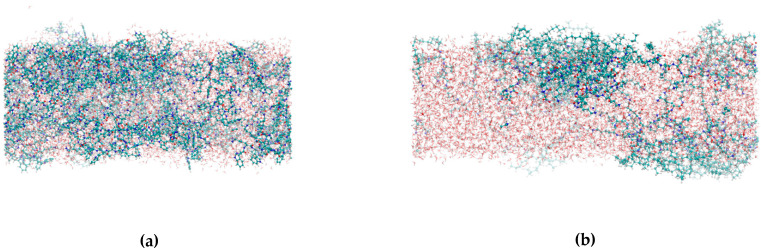

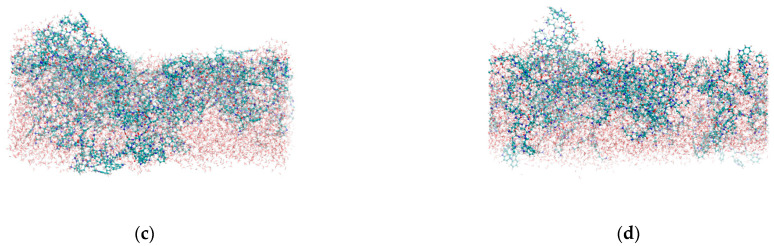
The structures of the peptide-water thin layers after the MD simulations. Peptide P2 system (**a**), P4 (**b**), P5 (**c**) and P6 (**d**).

The investigated systems after the MD simulations are shown in Figure 10a–d. It can be seen that the peptides tended to form a monolayer at the water–vacuum interface. This effect was mostly visible for the P4 and P5 peptides. For the P4 it can be explained by the presence of the aliphatic chains, which were of the hydophobic character, and they tend to be oriented away from the water layer. It is clearly visible in Figure 10b and is consistent with the smallest surface tension value of 934.92 bar × nm. The P5 peptide also contained non-polar side chains which contributed to this effect; however for this system there was also a steric effect playing a role. It can be observed as a bulge on top of the water film in Figure 10c. This bulge was an agglomeration of the peptide molecules, and within the time of the simulation it remained stable. This rigid structure was formed due to the interaction between the peptide molecules. It was consistent with the smallest observed diffusion coefficient of 0.0765 × 10^−5^ cm^2^/s.

Slightly different behaviour was observed for P6 peptide system. This peptide is characterized by a presence of two groups of amino acids: 3 non-polar tryptophans were connected by a peptide bond to 5 branched lysine amino acids, which were electrostatically charged. These were able to penetrate the water film much easier. It can be observed that the peptide was not located at the water–vacuum interface, but deeper in the water film, despite the presence of the non-polar tryptophan amino acids. This observation was consistent with the highest observed diffusion coefficient among the investigated systems—0.2763 × 10^−5^ cm^2^/s (Table 3).

P2 system was characterized by the presence of intertwined lysine and tryptophan amino acids. That made it different from the P6, because the polar and non-polar parts of the molecule were not separated from one another. This peptide was penetrating the water film much deeper than any other investigated system and should be considered the most hydrophilic one among the investigated systems. In addition, only for this system, it can be observed that the same amount of peptide resided at the bottom part of the water layer as at the top layer, what indicates that peptide molecules can reposition during the MD run. Still, some symptoms of the preference of the surface could be observed—the peptide did not reside within the bulk of the water layer, but rather near the surface.

## 3. Discussion

The desired direction of research is the search for compounds with high therapeutic potential and synergy with currently used antibiotics. At present, antibacterial peptides and lipopeptides are an increasingly studied group of compounds [20,21,22]. Their diverse structure results in different physicochemical properties, e.g., relative chemical and physical stability, variety of sequences and forms, ease of functionalization with (bio)molecules and the possibility of synthesizing them in large quantities [23,24]. In our research, we selected four peptides P1 (WKWK)_2_-KWKWK-NH_2_, (C12)_2_-KKKK-NH_2_, (KWK)_2_-KWWW- NH_2_ and (KK)_2_-KWWW-NH_2_ out of six that showed anti-acne properties.

**Figure 11:**
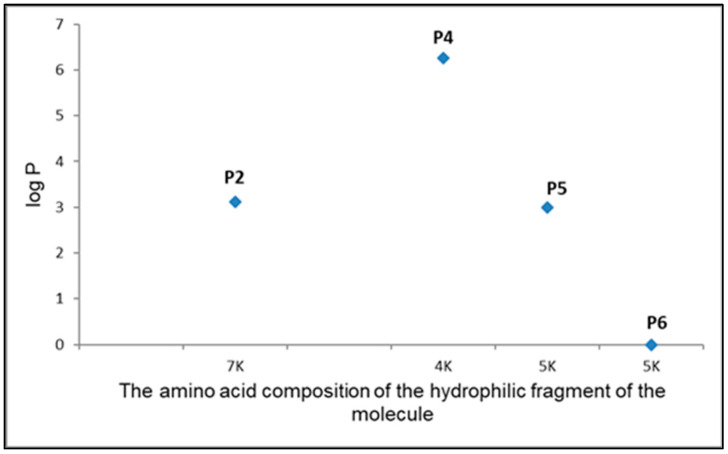
Chart of the dependence of the amino acid composition of the polar fragment of the molecule on the log P. Marking on the axis of arguments mean K-lysine.

Substances with a high logP value (i.e., substances with high lipophilicity) penetrate very easily into the stratum corneum, but their penetration into the living layers of the epidermis is already inhibited due to the hydrophilic nature of these layers. The opposite is true for hydrophilic (low logP) substances. They can easily diffuse through the living layers of the epidermis and dermis but are unable to overcome the lipophilic barrier of the stratum corneum. To sum up, substances with medium lipophilicity, showing significant solubility in both water and lipids, with a maximum for the logP value of 1.0–3.0, will have the optimal penetration ability [25,26]. In our case, these requirements would be met by P2, P5 and P6 (Figure 11). To facilitate administration of drugs through skin, penetration enhancers are routinely employed [27], which would be correct when using the P4 compound with high lipophilicity and proven anti-acne and anti-fungal properties.

These peptides showed high thermal stability. From the results obtained with the TG method, we know that the initial significant mass loss occurred only at about 230 °C and 350 °C, while in the case of P4 additionally at 406 °C. Changes between 200 and 500 °C were related to the degradation of the sample, namely progressive deamination, decarboxylation, and depolymerization resulting from the breaking of polypeptide bonds [28]. Different thermal behavior of the tested compounds results from their structure, i.e., different sequence of amino acids. In addition, the peptide molecule must gain a certain degree of freedom for any degradation reaction to take place [29].

The monolayer compressibility factor allowed us to characterize the state of monolayers, phase transitions, and take different values depending on the phase in which it is located. For the gaseous state, it has a value from 0 to 12.5 mN/m [30]. For the expanded liquid (LE) it takes values from 12.5 to 50 mN/m, for the condensed liquid phase (LC) from 50 to 250 mN/m, and for the solid phase above 250 mN/m [31,32]. Analysis of the compressibility factor of individual peptides indicated that all formed peptide monolayers are in the expanded liquid phase. Their maximum compressibility factor was lower than 50 mN/m. Its highest value of 42.7 mN/m was achieved by a monolayer made of P4. The monolayer formed from P2 was close to the gas phase, and actually on the border between the gas phase and the expanded liquid. The same was true for the P6 monolayer, except that the compressibility factor of 14.6 mN/m below 12.1 Å^2^/particle decreased towards smaller areas per particle and at about 4 Å^2^/particle did not exceed 10 Å^2^/particle. Mixtures of compounds showed lower values of the compressibility factor compared to their single components. If the compression and decompression isotherms coincide, it means that the monolayer has reached a stable state, and the composition of the monolayer remains the same as in the P2–P5 system (1:1). When the next hysteresis cycle moves towards smaller surfaces per molecule, it may indicate a greater packing of molecules, as in the P2–P5 system, or perhaps a transition of molecules to the aqueous subphase, e.g., P2–P6 system [33] These conclusions were supported by the MD simulation, which showed the influence of the monolayer-forming peptide structure on the structure of formed monolayer. In the case of the monolayer formed with P4, non-polar side chains played an important role, and the same is true for P5, except that a spherical effect was observed here. A slightly different behavior was observed for the P6 and P2 peptide systems, where the order in which the amino acids occur had an influence here [34].

Based on the compression isotherms of the tested peptides and their systems in various proportions, it can be concluded that all of them form Langmuir monolayers and thus the tested compounds could be tested in the form of anti-acne bacteria thin films and in the future applied as anti-bacterial systems based on the monolayers. By using systems with different proportions of P2, P4, P5, P6, we could obtain a system with the expected monolayers physical properties.

## 4. Materials and Methods

### 4.1. Synthesis and Characterization of the Peptides

#### 4.1.1. Preparation of the Peptides

The Rink amide AM resin and the amino acids Fmoc-Lys(Boc)-OH. Fmoc-Lys(Fmoc)-OH. Fmoc-Arg(Pbf)-OH and Fmoc-Trp(Boc)-OH were obtained from Iris Biotech (Marktredwitz, Germany). The dodecanoic acid coupling reagents and solvents: N,N-dimethyl formamide (DMF), dichloromethane (DCM), 1-hydroxybenzotriazole (HOBt), trifluoroacetic acid (TFA) and acetonitrile (ACN) were purchased from Merck (Darmstadt, Germany).

The peptide sequences were de novo designed to present positive charge by the incorporation of arginine or lysine residues. Tryptophan residues and dodecanoic fatty acid were used to provide the ability to insert into bacterial membranes. The peptide compounds were manually synthesized by Fmoc solid phase peptide synthesis using the Rink amide AM resin (100–200 mesh; loading 0.48 mmol/g). The coupling reaction of the amino acids was made with the activators DIC and HOBt with three times molar excess of each amino acid and activator. dissolved in DMF/DCM (1:1; *v*/*v*) mixture. Deprotection was carried out with 20% (*v*/*v*) of piperidine in DMF. De-anchoring of the peptides from the resin was achieved with TFA/TIS/H2O mixture in a volume ratio (95:2.5:2.5).

#### 4.1.2. Purity and Structure of the Peptides

Purity of the lipopeptides was analyzed by reverse phase high performance liquid chromatography (RP-HPLC) in Shimadzu Nexera chromatograph with a DAD detector at 214 nm fitted with a Eurospher (100 × 4.6 mm) columns (Knauer, Berlin, Germany) using ACN:TFA (0.1%) and H2O:TFA (0.1%) as the mobile phase. The identity of lipopeptides was verified by matrix-assisted laser desorption time-of-flight (MALDI-TOF) spectrometry on MALDI-TOF/TOF 5800 (Sciex, IL, USA). USA). The peptides with identity confirmed via MS spectra were freeze-dried (Christ, Hannover, Germany) and stored as dry powder at −20 °C.

### 4.2. Thermogravimetric TG

Thermogravimetric analysis of the tested peptides was carried out using a thermobalance (TG 209 F1 Libra. Netzsch. Selb, Germany) with an automatic sample changer (ASC. Netzsch. Selb, Germany). The thermograms were scanned at a constant heating rate of 5 K/min in the temperature range from 10 to 300 °C.

### 4.3. DSC Investigation

DSC studies were performed using a differential scanning calorimeter (DSC 214 Polyma. Netzsch. Selb, Germany). Samples of 3–5 mg were measured in sealed aluminum pans under a nitrogen atmosphere. with a flow rate of 50 mL/min.

### 4.4. Langmuir Films

The Langmuir–Wilhelmy trough manufactured by Kibron Microtrough X manufactured in Helsinki (Finland) together with the attached computer software Filmware X 4.0. was used to study the monolayers formed by the tested peptides. The balance consists of a tetrafluoroethylene (Teflon) tray measuring 23.7 cm long and 7.9 cm wide, two movable Teflon barriers and a wire (used instead of a Wilhelmy plate) weighing 48.2 mg and 0.5 mm in diameter made of platinum, which ensures a negligible contact angle during the measurement. The barriers moved at a speed of 10 mm/min.

#### 4.4.1. Preparation P2, P4–P6 Solutions Forming a Monolayer

Solutions of the tested compounds were prepared by dissolving the previously weighed substance in a solvent solution, which was a mixture of chloroform and methanol solution in the ratio of 3:1. First, compression isotherms were measured for different dilutions to select one appropriate concentration for all peptides. The number of particles deposited on the subphase [35] for successive dilutions for successive dilutions (D1–D6) was: 2.14·10^16^ (D1), 1.07·10^16^ (D2), 5.35·10^15^ (D3) 2.68·10^15^ (D4), 1.34·10^15^ (D5), 6.70·10^14^ (D6) molecules. In the case of mixed systems, the area occupied by a given monolayer can be presented as the sum of the areas of individual components. This situation is presented by the Equation:

*A*_12_ = *A*_1_*x*_1_ + *A*_2_*x*_2_

where: *A*_12_—surface area per single molecule in mixed monolayers; *A*_1_ and *A*_2_—values of the areas occupied by individual single pure components; *x*_1_ and *x*_2_—molar ratio in which single pure components occur.

#### 4.4.2. Hysteresis

Isotherm compression–decompression was used analogously to measure isotherm compression. The same speed of moving and spreading the barrier was used: 10 mm/min. However, in this case the recorded measurement was for 3 loops. In the Filmware X 4.0 program (Kibron, Helsinki, Finland), the measurement range was determined on the basis of isotherm compression.

#### 4.4.3. Compressibility Coefficient of the Monolayer

The monolayer compressibility coefficient made it possible to determine the mechanical properties of the monolayer [36,37]:

$$C_{S}^{-1}=-A\left(\frac{d\pi }{\mathrm{dA}}\right)$$

where C_S_^−1^—compressibility factor [mN/m], A—surface area per molecule (Å^2^/molecule), π—surface pressure (mN/m).

### 4.5. Molecular Dynamics Simulations

The systems consisting of the individual peptides in the thin water film have been simulated using the Molecular Dynamics (MD) simulations. Each investigated system has been constructed using Packmol software [38] to contain 50 molecules of the peptide and 9000 molecules of water. The peptide molecules have been placed on top of the water film. In order to maintain charge neutrality, a number of water molecules corresponding to the number of positively charged groups in the peptide was replaced with Cl-anions. The system was made periodic in all 3 directions, with the box orthogonal box size of 10 nm × 10 nm × 20 nm.

We have used the CHARMM potential version 27 [39] to describe the inter- and intramolecular interactions. The parameters were used “without modifications” for P2, P5 and P6. For the aliphatic chains of P2 we have used the same parameters as in the aliphatic groups in leucine and isoleucine. The interactions between the carbonyl groups and the amine group of lysine were treated identically as all other peptide bonds. The cutoff for the vdW and Coulomb interactions was set to 1.0 nm. The electrostatic interactions in the periodic boundary conditions have been described by means of the Ewald summation method [40]. All simulations have been carried out using the GROMACS suite of codes [41,42,43].

Prior to the MD simulations, the systems geometry has been optimized to reach the forces on each atom no greater than 1000 kJ/mol/nm. Then, a 100 ps long equilibration runs have been carried out, followed by 1 ns production run. The timestep was set to 1 fs in both types of MD runs. The hydrogen bonds were constrained using the LINCS algorithm [44]. The temperature was set to 300 K, and was controlled using the velocity rescaling, modified Berendsen thermostat [45].

## 5. Conclusions

The obtained results indicate that the peptides (WKWK)_2_-KWKWK-NH_2_, (C12)_2_-KKKK-NH_2_, (KWK)_2_-KWWW-NH_2_, (KK)_2_-KWWW-NH_2_ are thermally stable and tend to form a monolayer at the water-vacuum interface. This effect is most pronounced for the P4 and P5 peptides. In the case of P4, this can be explained by the presence of aliphatic chains that are hydrophobic and oriented away from the water layer, while P5 also plays a role in the steric effect. Calculations of the logP parameter, compressibility coefficient, molecular dynamics simulation, and compression isotherms indicate that P2 and P6 penetrate much deeper into the water layer than P4 and P5. The peptide structure strongly influences the monolayer properties. The amino acids arrangement in molecule, as well as the proportion of respective polar groups is an important factor in superficial activity of assessed peptides.

## Figures and Tables

**Table 1:** Tmax, weight loss, and residual weight of assessed peptides P2, P4–P6 according to performed TD and DTG plots.

Peptide	T_max_ [°C]	Loss of Mass [%]	Residual Mass [%]
P2	- 145.0 229.5 352.2	1.87 2.11 23.80 60.52	11.50
P4	230.2 359.0 406.3	25.12 56.08 18.80	0.00
P5	148.2 228.6 351.2	2.79 22.37 58.31	16.42
P6	228.5 351.3	30.01 56.40	13.48

**Table 2:** DSC peaks (°C) of P2, P4–P6 peptides obtained.

**Temperature [°C]**	**P2**	**P4**	**P5**	**P6**
	45.3 ↑ 101.9 ↓ 138.3 ↑	61.8 ↓ 86.0 ↑ 108.5 ↓ 151.1 ↑	42.8 ↑ 115.5 ↓ 140.8 ↑	48.4 ↑ 109.6 ↓ 126.9 ↑

↓—exothermic proces, ↑—endothermic process.

**Table 3:** The values of the diffusion coefficients of the peptide molecules in the water films and the surface tensions in the systems.

Peptide	P2	P4	P5	P6
D_coeff_ (cm^2^/s)	0.1067 × 10^−5^	0.0878 × 10^−5^	0.0765 × 10^−5^	0.2763 × 10^−5^
γ (bar × nm)	1116.22	934.92	1003.66	1005.38

Where D_coeff_ is coefficient of diffusion and γ is surface tension value.

## Data Availability

The data are available by the authors.
